# Refractive, visual, and subjective quality of vision outcomes for very high myopia LASIK from − 10.00 to − 13.50 diopters

**DOI:** 10.1186/s12886-020-01481-2

**Published:** 2020-06-17

**Authors:** Avi Wallerstein, Joseph Wai Keung Kam, Mathieu Gauvin, Eser Adiguzel, Mounir Bashour, Ananda Kalevar, Mark Cohen

**Affiliations:** 1 Department of Ophthalmology, McGill University, Montreal, QC Canada; 2 LASIK MD, 1250 Rene-Levesque Blvd W, MD Level, Montreal, QC H3B 4W8 Canada; 3 Department of Ophthalmology, University of Sherbrooke, Sherbrooke, QC Canada

**Keywords:** LASIK, Very high myopia, High myopia, Quality of vision

## Abstract

**Background:**

To evaluate laser-assisted in situ keratomileusis (LASIK) outcomes, subjective quality of vision (QoV) and patient satisfaction in eyes with very high myopia (VHM) above − 10.00 diopters (D).

**Methods:**

Consecutive myopic and myopic-astigmatism eyes with spherical equivalent (SEQ) ranging between − 10.00 to − 13.50 D underwent LASIK with the WaveLight® Allegretto Wave® Eye-Q 400 Hz excimer laser. Treatment accuracy, efficacy, safety, stability, cylinder vectors, and higher-order aberrations were evaluated, together with subjective QoV and night vision disturbances (NVDs).

**Results:**

114 eyes had a preoperative SEQ of − 11.02 ± 0.81 D, with a median follow-up of 24 months. A total of 72, 84, and 94% of eyes were within ± 0.50, ± 0.75 and ± 1.00 D of intended SEQ (*R*^2^ = 0.71). The efficacy index was 0.93 ± 0.20, with 51 and 81% of eyes achieving 20/20 and 20/25. The astigmatism correction index was 0.95 ± 0.33. The safety index was 1.05 ± 0.12. The average myopic regression was − 0.51 ± 0.38 D. Preoperative QoV scores improved significantly postoperatively (7.5 ± 0.8 vs. 9.1 ± 0.7; *P* <  0.001), with less NVDs (*P <  0.001*). Total, spherical and coma root mean square (RMS) postoperative ocular higher-order aberrations were 1.07 ± 0.34, 0.67 ± 0.25, and 0.70 ± 0.40 μm.

**Conclusions:**

Very high myopia LASIK between − 10.00 to − 13.50 D is safe and results in good visual outcomes, with high patient satisfaction and a significant improvement in patient-reported QoV after surgery. Appropriately selected patients within this very high myopia group can be included as LASIK candidates.

## Background

Many surgeons are apprehensive in performing laser-assisted in situ keratomileusis (LASIK) in patients with very high levels of myopia, greater than − 10.00 diopters (D), due to concerns regarding outcomes and safety. Many of these concerns originate from older generation laser technology and include losing corrected distance visual acuity (CDVA) and inducing poor quality of vision (QoV) due to small effective optical zone size, increased spherical aberration, flat keratometry, and higher microstriae rates. There are also concerns related to under correction, higher regression rates, insufficient corneal tissue to perform both an initial treatment and enhancement, and increased ectasia risk. Photorefractive keratectomy (PRK) and Phakic intra-ocular lenses have also been promoted for these cases [1, 2]. Newer, advanced laser platforms can be used for high myopic correction, but the effectiveness and safety profile has not been adequately characterized in very high myopia (VHM) eyes with spherical equivalent (SEQ) exclusively above − 10.00 D, with a limited number of eyes in the literature. This study undertook a detailed outcomes analysis of a large sample of LASIK eyes with VHM from − 10.00 to − 13.50 D using a fast repetition scanning small spot excimer laser. This study also reports on postoperative ocular higher-order aberrations with patient-reported subjective QoV and satisfaction.

## Material and methods

### Protocol and patient selection

This retrospective study reviewed patients who presented for corneal refractive surgery with VHM, defined as SEQ of − 10.00 D or greater. 11 surgeons working in 9 clinics performed surgery. All locations were part of the same Canadian corporate refractive surgery practice with standardized techniques, protocols, nomograms, and equipment. As part of credentialing, all surgeons received the same training course consisting of an observership and proctorship, and attended a yearly didactic teaching conference. All surgeons had proprietary teaching manuals readily available as well as a peer consult group to communicate with on an as needed basis regarding patient care.

Eyes up to − 13.50 D of SEQ were screened. Normal corneal topography excluding signs of keratoconus, and other standard inclusion criteria for LASIK were required. Excluded were eyes with a calculated RSB of less than 280 μm, patients with retinal pathology effecting corrected distance visual acuity (CDVA), peripheral retinal changes requiring laser photocoagulation, those with lens opacities, and patients who were contact lens tolerant. Only primary surgery outcomes are reported without enhancements. In addition to the customary LASIK consent forms, patients were required to attest that a second procedure may not be possible with under correction or regression, and with an understanding of the potential higher risk of post-LASIK ectasia. Patients also consented to the use of their non-identifying clinical data for research purposes. The study adhered to the tenets of the Declaration of Helsinki and was approved by the Ethics Review Board.

### Preoperative assessment

A standard preoperative refractive surgery ocular exam was completed with attention to perform a thorough manifest refraction. The vertex distance was measured and set to 12 mm in all eyes.

### Surgical technique

LASIK was performed under topical anesthesia (Alcaine Drops, Alcon). All surgeons followed the same previously described standardized technique [3–5], using the same equipment and identical nomograms. Custom-Q® treatment software (F-CAT) was used on the WaveLight® Allegretto Wave® Eye-Q 400 Hz excimer laser. The Intralase femtosecond laser iFS (Abbott Medical Optics, Inc., Santa Clara, CA) or the Hansatome Microkeratome (Bausch & Lomb, Rochester, NY) with Z15 or Z16 head in combination with an 8.5- or 9.5-mm suction ring were used to create the corneal flaps. There were no statistical differences in preoperative and postoperative variables between Hansatome and femtosecond flaps and data were pulled together. Emmetropia was the target postoperative refraction for all treatments. Optical zone size was decreased in certain eyes below 6.5 mm to a minimum of 6.0 mm to save corneal tissue. Additional surgical technique details were published elsewhere [3–5]. A standardized postoperative regimen of antibiotics and steroids was used [6].

### Data and statistical analysis

Preoperative exams and collected data included medical and ocular history, manifest refraction (MR) sphere, cylinder and axis, uncorrected distance visual acuity (UDVA) and CDVA, slit-lamp biomicroscopy, applanation tonometry, ultrasonic pachymetry, fundus exam, and Orbscan IIz corneal topography. Postoperative follow-up exams were performed at 1 day, 1, 3 and 6 months, as well as later time points, and included MR sphere, cylinder and axis, UDVA, CDVA, slit-lamp biomicroscopy, and Orbscan IIz topography. Postoperative Zywave aberrometry on a 6.5 mm zone was assessed at 6 months and later time. Standardized satisfaction and subjective QoV questionnaire based on McAlinden and colleagues [7]. Validated survey was randomly given to a third of patients. Accuracy, efficacy, refractive astigmatism, cylinder vector analysis, safety, stability, ocular higher-order aberrations (HOA), subjective QoV, and complications are presented. Data were reported as mean ± standard deviation (SD). Pearson’s correlation tests were used to assess the relationship between selected pairs of variables. Statistical analyses were carried out in MATLAB R2019a software (Mathworks, Natick, MA, USA) with the level of significance set at *p* <  0.05.

## Results

A total of 114 eyes from 78 patients who underwent LASIK are reported. Of the subjects, 62.8% were female and 37.2% were male. The average age was 34.2 ± 8.9 years (range: 19 to 55 years). Table 1 reports preoperative and intra-operative parameters. Mean preoperative SEQ was − 11.02 ± 0.81 D (maximum of − 13.50 D), and the average postoperative follow-up time was of 27.4 ± 12.8 months, with a median of 24.1 months. The average flap thickness was 117.2 ± 21.3 μm. The average ablation depth was 137.7 ± 10.8 μm. The percent tissue altered (PTA) was 42.6 ± 2.4%. The average minimal keratometry postop was 36.1 ± 1.7 D (32.5 to 39.6).

**Table 1:** Preoperative and Intra-Operative Parameters

	Mean ± SD	Range
**Preoperative variables**		
Sphere (D)	−10.33 ± 0.82	− 8.75 to − 12.50
Cylinder (D)	−1.20 ± 0.87	0.00 to − 4.25
Spherical equivalent (D)	−11.02 ± 0.81	−10.00 to − 13.50
Central corneal thickness (μm)	580.3 ± 25.9	519 to 656
Pupil diameter (mm)	6.9 ± 0.9	4.4 to 8.6
Minimum keratometry (D)	43.5 ± 1.8	39.6 to 49.0
Maximum keratometry (D)	44.8 ± 1.7	41.3 to 50.2
**Intra-operative variables**		
Average flap thickness (μm)	117.2 ± 21.3	90.0 to 141.4
Average ablation (μm)	137.7 ± 10.8	115.6 to 164.1
Average % of cornea ablated	42.6 ± 2.4	38.4 to 47.7
Residual stromal bed (μm)	314.3 ± 62.3	280.0 to 381.5
Optical zone (mm)	6.5 ± 0.1	6.0 to 6.5

### Accuracy

**Fig. 1:**
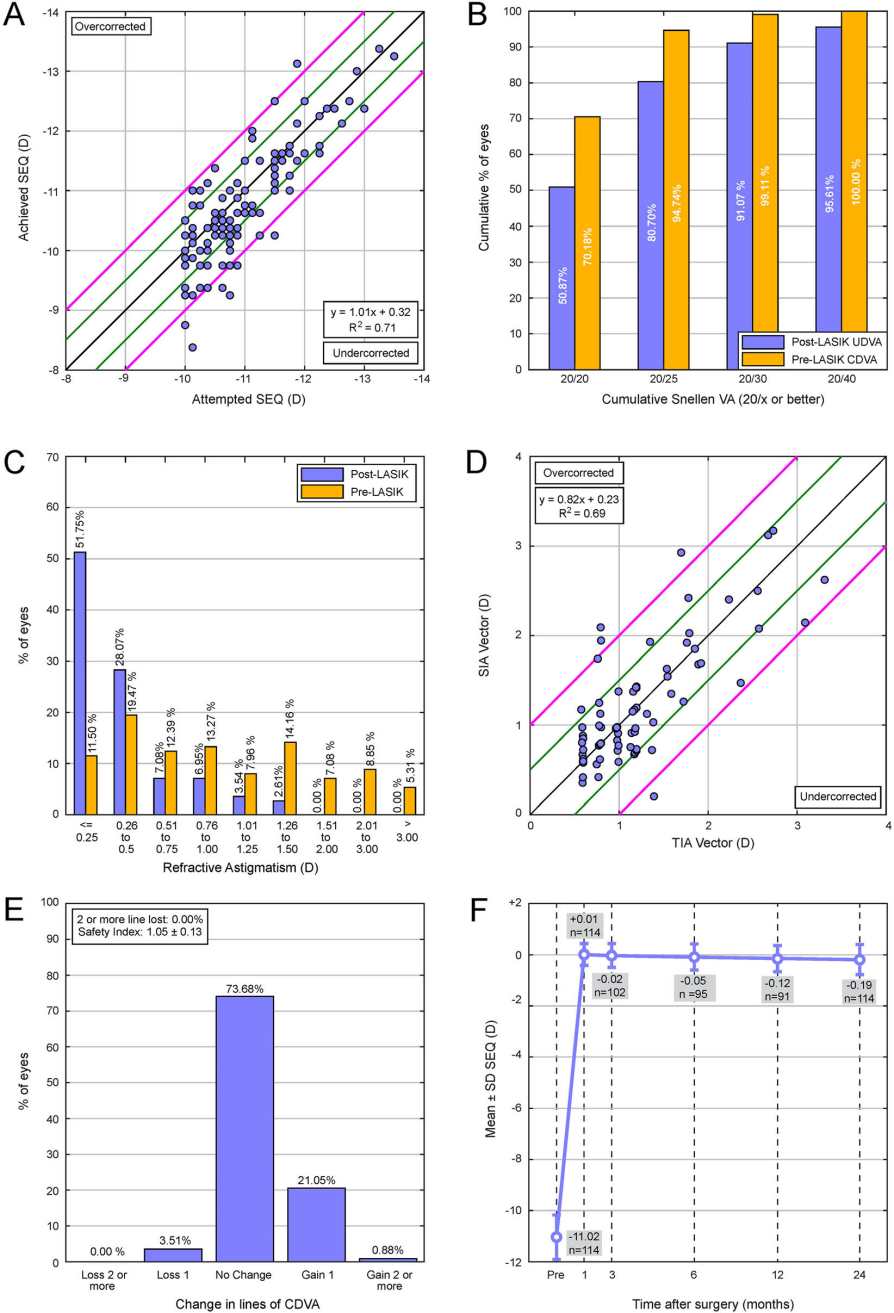
**a** Attempted versus achieved spherical equivalent (SEQ). Blue line indicates attempted = achieved. Green lines indicate ±0.50 D. Pink lines indicate ±1.00 D. **b** Postoperative cumulative Snellen UDVA compared with pre-LASIK CDVA. **c** Postoperative refractive astigmatism accuracy compared with pre-LASIK. **d** TIA vector vs. SIA vector for pre- and post-LASIK. Blue line indicates TIA = SIA, green lines indicate ±0.50D, pink lines indicate ±1.00D. **e** Postoperative change in Snellen lines of CDVA compared with pre-LASIK CDVA. **f** Spherical equivalent (SEQ) stability from before LASIK to 1, 3, 6 and 24 months after LASIK

At 24 months, the scatterplot of attempted versus achieved refractive correction revealed a predictable procedure (*R*^2^ = 0.71; Fig. 1a), with 51.7, 71.9, 84.2, and 93.8% being within ± 0.25 D, ± 0.50 D, ± 0.75 D, and ± 1.00 D, of SEQ target. More specifically, 7.9% were overcorrected (SEQ ≥ + 0.50 D), 20.2% were undercorrected (SEQ ≤ − 0.50 D), 71.9% were within ± 0.50 D.

### Efficacy

At the last follow-up (24 months), 50.9, 80.7, and 95.6% had postoperative cumulative UDVA of 20/20, 20/25, and 20/40 or better (Fig. 1b), compared to 70.2, 94.7, and 100% for the preoperative cumulative CDVA (Fig. 1b). A total of 86.5% were within 1 line of CDVA. The efficacy index was 0.93 ± 0.20. A total of 79.5% of patients achieved a bilateral UDVA of 20/20.

### Refractive astigmatism and cylinder vector

At 24 months, 51.8, 79.8, and 93.9% were within 0.25 D, 0.50 D, and 1.00 D of intended plano cylinder postoperatively. Mean preoperative cylinder was − 1.20 ± 0.87, compared to − 0.41 ± 0.38 postoperatively (*P <  0.001*). Alpins astigmatism vector analysis revealed a correction index (CI) of 0.95 ± 0.33.

### Safety

Postoperatively, 3.5% lost 1 line of CDVA (3 eyes from 20/20 to 20/25; 1 eye from 20/25 to 20/30), 73.7 were unchanged, and 21.9% gained lines of vision (Fig. 1e). The safety index was 1.05 ± 0.12.

### Stability

Postoperative SEQ was stable at the 1 month 3, 6, and 24-month time points. (Fig. 1f), with a non-significant trend toward a decrease in SEQ at the last follow-up (*P = 0.11*). The final SEQ, obtained at 24 months postoperatively, was − 0.19 ± 0.46 D. From 1 month to 24 months, the average amount of myopic regression was of − 0.51 D ± 0.38 D (*P <  0.001*), while 25.4% had myopic regression greater or equal to 0.50 D, 8.7% had a hyperopic shift greater or equal to 0.50 D, and 52.6% did not have a SEQ change greater than ± 0.25 D. There was no significant correlation between the absolute amount of SEQ change between 1 and 24 months and the preoperative SEQ nor the residual stromal bed (RSB) (*R = 0.18*; *P = 0.13*).

### Ocular higher-order aberrations

Total root mean square (RMS) postoperative HOAs were 1.07 ± 0.34 μm. Coma was 0.70 ± 0.40 μm, and spherical aberration was 0.67 ± 0.25 μm. There was no significant correlation between postoperative SEQ and total RMS postoperative HOAs (*R = 0.07*; *P = 0.67*) and total RMS postoperative coma (*R = 0.17; P = 0.54*).

### Subjective patient-reported outcomes

All randomly selected patients completing the postoperative questionnaire rated the surgery as improving their overall QoV compared to preoperative spectacle-corrected QoV, with 90.5% reporting significantly better, and 9.5% as moderately better. Postoperative uncorrected QoV was rated significantly higher than preoperative spectacle-corrected QoV (9.1 ± 0.7 versus 7.5 ± 0.8; *P <  0.001*). There was a statistically significant inverse correlation between uncorrected QoV rating and final refractive error (*R = − 0.47*, *P = 0.02*), but no correlation between uncorrected QoV and total RMS postoperative HOAs (*R = 0.04*; *P = 0.79*), total RMS postoperative coma (*R = 0.05; P = 0.85*), or postoperative keratometry (*R = − 0.22*; *P = 0.332*). Night vision disturbances (NVDs) were reported significantly less often postoperatively (uncorrected vision), compared to spectacle-corrected preoperative vision (Glare: *P = 0.016*; Haloes: *P = 0.052*; and Starbursts: *P <  0.001*; Table 2). Similar statistically significant improvements were seen in other visual phenomena (Table 2).

**Table 2:** Preoperative and postoperative subjective quality of vision and night vision disturbances

	Spectacle-corrected vision (Preop)	Uncorrected vision (Postop)	***P***-value
**Quality of vision (Mean score)**			
QoV	7.5 ± 0.8	9.1 ± 0.7	**< 0.001**
**Night vision disturbances (Mean score)**			
Glare	1.5 ± 2.9	0.5 ± 1.0	**0.016**
Haloes	2.0 ± 3.6	0.9 ± 1.6	0.052
Startbursts	2.2 ± 3.9	0.5 ± 1.0	**< 0.001**
**Other vision disturbances (Mean score)**			
Hazy vision	0.6 ± 1.0	0.2 ± 0.4	**< 0.001**
Blurred vision	3.3 ± 4.2	0.4 ± 0.5	**0.003**
Distorsion	0.8 ± 2.7	0.0 ± 0.0	**0.041**
Multiple images	0.4 ± 1.0	0.1 ± 0.3	**0.044**
Fluctuation	0.6 ± 1.6	0.3 ± 0.4	0.164
Focusing	2.9 ± 3.6	0.5 ± 0.5	**< 0.001**
Depth perception	1.4 ± 2.8	0.1 ± 0.3	**0.001**

### Complications

There were no intra-operative flap complications. Microstriae outside the visual axis were noted in 11.9% of eyes. Two striae eyes were clinically significant and needed re-lift and irrigation. There were no topographic findings to suggest postoperative ectasia as of the last follow-up visit, and no eyes presented with a retinal detachment.

## Discussion

LASIK outcomes are reported in moderate to high myopia [8–13], but data for eyes with VHM greater than − 10.00 D is sparse. Even the few eyes reported are grouped with lower levels of myopia, with short follow-ups, or retreatments as part of the outcome analyses (Table 3). In the past 10 years, only four published studies reported on LASIK outcomes with an average SEQ above − 10.00 D [14–17]. Only one report by Artini and colleagues [14] used a fast repetition small scanning spot excimer laser (Alcon EX-500) with an average preoperative SEQ of − 11.40 D, but with a short 2 month follow-up (Table 3). At 2 months 69.9% of 99 eyes were within ±0.50 D of intended SEQ compared to a similar 71.9% at 24 months in this study. Lindbohm and colleagues [16] used the VISX Star S2 laser to treat myopes that had an average preoperative SEQ of − 11.70 D. Six months postoperatively 40% of eyes were within ±1.00 D of intended correction, and 8% of eyes achieved a UDVA of 20/20. Rosman and colleagues [17] used the VISX 20/20 excimer laser to treat VHM patients that had an average preoperative SEQ of − 12.81 D. At the 10 year follow up, 42.5% of the eyes within ± 1.00 D of intended correction, 45.5% of eyes achieved a UDVA of 20/40, and the mean total regression was 1.49 ± 2.17 D. While the preoperative SEQ were similar (− 11.70 D versus − 12.81 versus − 11.02 D), the current study’s and Artini’s outcomes are significantly better, suggesting that small scanning spot lasers yield better accuracy and efficacy than older generation lasers in VHM. This is likely due to the wavefront-optimized aspheric ablation profile with larger effective optical zones, and a faster eye tracker. Oruçoğlu and colleagues used the Keracor Technolas excimer laser (50 Hz scanning spot) to treat extreme high-myopia, with an average preoperative SEQ of − 21.70 D [15]. With such a high level of attempted correction, their outcomes were much less favorable. A direct comparison to this study is limited.

**Table 3:** Literature Review of LVC for High and Very High Myopia

Author (year)	N (eyes)	Laser	Preop SEQ	F/U	Postop SEQ	Within ± 0.50 D	Within ± 1.00 D	UDVA 20/20	Loss 1 line of CDVA	Loss 2 lines of CDVA	Retreats
**Very High Myopia**^**a**^											
Wallerstein (2020)	114	Alcon 400 Hz	−11.02 ± 0.81 D	24 months	−0.20 ± 0.65 D	71.9%	93.8%	50.9%	3.5%	0%	No
Artini (2018) [14]	99	Alcon 500 Hz	−11.40 D	2 months	0.00 D	69.9%	–	–	–	–	No
Oruçoğlu (2012) [15]	143	B&L Technolas	−21.7 ± 5.80 D	10–15 years	−6.09 ± 3.35 D	14%	–	–	10.3%	–	No
Lindbohm (2009) [16]	77	VISX Star S2	−11.70 D	5 years	−1.95 ± 1.45 D	–	40%	8.0%	7.7%	1.9%	Yes
Rosman (2010) [17]	114	VISX 20/20	−12.81 ± 1.64 D	10 years	−1.48 ± 1.99 D	28.0%	42.5%	–	–	6%	Yes
**High Myopia**^**b**^											
Vega-Estrada (2019) [18]	70	Schwind 500 Hz	−7.79 ± 1.38 D	5 years	−0.24 ± 0.57 D	62%	76%	59%	6%	0%	Yes
Artini (2018) [14]	219	Alcon 500 Hz	−8.00 D	2 months	0.00 D	96.1%	–	–	–	–	No
Xia (2018) [19]	65	Zeiss 500 Hz	- 8.05 ± 1.12 D	12 months	−0.43 ± 0.82 D	–	–	90.8	0%	0%	No
Low (2018) [20]	50	Alcon 400 Hz	−9.56 ± 0.86 D	3.6 months	0.26 ± 0.34 D	84.0%	100%	66.0%	0%	2%	No
Niparugs (2018) [21]	93	Alcon 500 Hz	−7.83 ± 1.18 D	12 months	−0.14 ± 0.30 D	83.7%	96.7%	85.6%	12.9%	0%	No
Liu (2017) [22]	104	Alcon 400 Hz	−9.64 D	12 months	0.13 ± 0.04	91%	95%	85.0%	0%	1.9%	Yes
Ikeda (2017) [23]	68	VISX Star S2	−6.70 ± 2.52	12 years	−0.74 ± 0.99 D	53%	75%	43.0%	13%	5%	No
Reinstein (2016) [24]	479	Zeiss 250 Hz	−9.39 ± 1.22 D	17 months^c^	−0.39 ± 0.6 D	55.0%	81%	75.0%	2.9%	0%	No^d^
Hashemi (2016) [25, 26]	60	Alcon 500 Hz	−8.37 D	18 months	−0.24 ± 0.6 D	75.0%	100%	75.0%	–	–	No
Ide (2014) [27]	346	APEX Plus	−6.42 ± 2.70	10 years	−0.67 ± 0.92 D	–	76.3%	52.0%	–	4.6%	Yes
Kanellopoulos (2013) [28]	116	Alcon 500 Hz	−7.67 D	6 months	−0.43 ± 0.09 D	84.0%	96.3%	90.5%	0%	0%	No
Alio (2011) [29]	51	Schwind 500 Hz	−8.66 D	6 months	−0.42 ± 0.82 D	69.0%	89.6%	58.8%	6.9%	0%	No
Stonecipher (2010) [30]	65	Alcon 400 Hz	−7.07 D	6 months	−0.56 ± 0.56 D	100%	100%	92.0%	**_**	0%	No

^a^SEQ > −10.00 D ^b^(SEQ > −6.00 D)

^c^Estimated average (230 eyes at 24 months + 221 eyes at 12 months + 27 eyes at 6 months = 17.4 months)

^d^This study also included outcomes after retreat. This table reports data before retreats

High myopia studies with lower SEQ than the current VHM study include Liu and colleagues [22], using the Alcon WaveLight® 400 Hz excimer laser. They had an average preoperative SEQ of − 9.64 D vs. − 11.02 D in the current study (Table 3). The percentage of eyes with a true preoperative SEQ above − 10.00 D is unknown and VHM eyes were grouped with eyes as low as − 8.00 D SEQ. They also included outcomes with retreatments, limiting the comparison of their more favorable 91 and 95% of eyes within 0.50 and 1.00 D of attempted SEQ correction, and 85% of eyes achieving UDVA of 20/20.

Using a 250 Hz 1 mm small spot size excimer laser (Zeiss Mel 80), Reinstein and colleagues reported outcomes in high myopes with an average preoperative SEQ of − 9.39 D [24] (Table 3). Review of the attempted vs. achieved SEQ graph shows that approximately 78% of eyes had a preoperative SEQ below − 10.00 D. The other 22% with true VHM were grouped with eyes of SEQ as low as − 7.50 D for analysis. They report 55 and 83% of eyes achieving 0.50 D and 1.00 D of attempted SEQ correction, less than the current study’s 72 and 94%. Efficacy was similar, with 85% of their eyes having a postoperative UDVA within 1 line of preoperative CDVA, versus 86.5% here. Their average postoperative SEQ was more myopic at − 0.39 D vs. − 0.19 D in the current study. The current study with higher levels of myopia (− 11.02 D vs. -9.39 D SEQ) indicates that a 400 Hz 1 mm spot size laser can achieve more favorable outcomes in VHM patients at 24 months.

Other high-myopia studies with less myopia than current study (average SEQ between − 6.46 and − 9.56 D; Table 3), using a variety of excimer lasers, reported a wide range of SEQ accuracy within ±0.50 D, varying between 53 and 100% (Table 3) with an average of 77.5%, slightly higher than the 71.9% reported in the current study. The large variability that exists in both accuracy and efficacy (Table 3) is related to the levels of myopia treated, the preoperative CDVA, the inclusion of retreated eyes and differences in: refraction techniques, laser models, nomograms, and follow-up time points. An overall comparison suggests that treatment for lower myopia groups is somewhat more precise than treatment for VHM eyes.

The average postoperative SEQ refraction at 6 and 24 months in this study was of − 0.05 and − 0.19 D, respectively (Fig. 1f), which is comparable or better than other lower myopia studies [9, 16, 23–32] (Table 3). A better measure of regression effect, than the traditionally reported postoperative SEQ, is the amount of myopic development postoperatively. There was a significant average trend towards myopic regression of − 0.51 D between 1 and 24 months (*P <  0.001*), which is small considering this VHM group. Patients with high myopia are more likely to regress, with regression being reported to be more pronounced between 3 and 12 months [15, 33–35]. There may be less regression here than expected due to newer aspheric larger ablation profiles (Custom-Q®), improving stability. A follow-up study at later time points would help determine longer term regression rates. A positive correlation was previously found between postoperative SEQ regression 15 years after myopic laser vision correction (LVC) surgery and both RSB and PTA [33]. In the current study, these correlations were not statistically significant at 2 years.

The safety index of 1.05 was comparable to lesser myopia studies (Table 3), suggesting that LVC in VHM has equivalent safety to that of high myopia [16, 25, 29, 36]. As reported elsewhere [6], the high-rate of microstriae (11.9%) can likely be attributed to the very deep ablations (mean 150 μm), although only two eyes with microstriae were deemed to be visually significant requiring intervention. This VHM spectacle-wearing group may be more tolerant of the aberrations caused by striae, as they may have been accustomed to visual phenomena with spectacle correction [37, 38].

The PTA was 42.6 ± 2.4%. PTA above 40% has been described as a risk factor for ectasia [39]. After a follow-up of 24 months there were no cases of ectasia in this high PTA group of eyes. These findings may support those of Saad and colleagues [40] who feel PTA may not be a useful risk predictor for ectasia. Longer term follow-up would be needed to further monitor for ectasia. These eyes, possibly at higher risk, should have more frequent sequential follow-up visits to monitor for early signs of post-LASIK ectasia, that can be treated with under-flap corneal collagen cross linking with the potential to preserve vision [41].

**Fig. 2:**
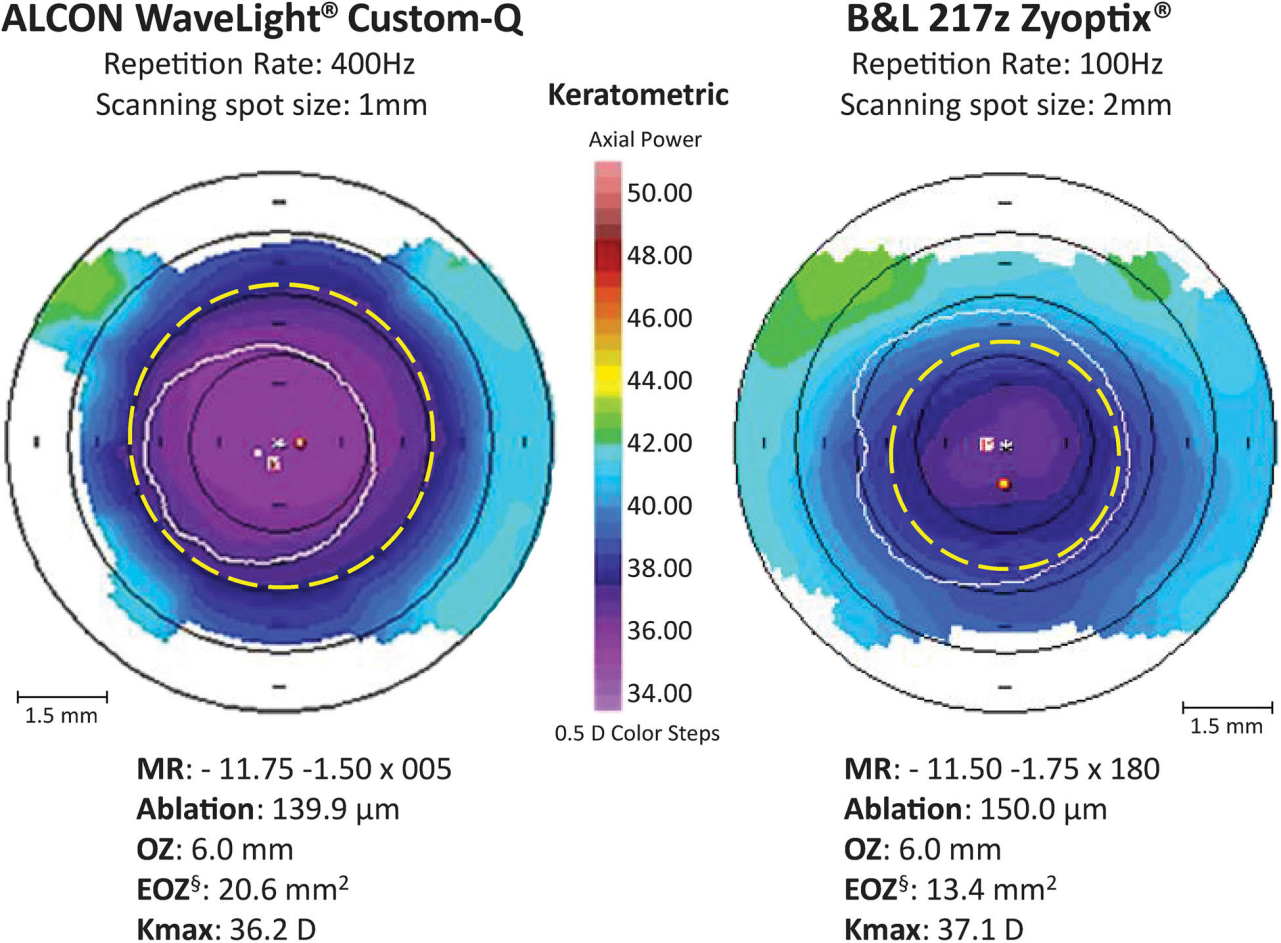
Comparison of topographic effective optical zone between a 400 Hz, l mm spot size, laser and a 100 Hz, 2 mm spot size, laser in very high myopia. ^§^EOZ defined as the circular area with an outer border 1 D (2 color steps) steeper than the central keratometry. The white circles show the pupil at the time of Orbscan acquisition and the yellow dashed circles outline the limit of the EOZ

The current laser platform also creates a larger effective optical zone size (EOZ) as a result of its newer ablation profile. A comparative − 12 D SEQ example using a 100 Hz 2 mm spot size laser versus a 400 Hz 1 mm laser is shown in Fig. 2. Note the significantly larger EOZ size with newer laser technology (20.6 mm^2^ vs. 13.4 mm^2^). Similarly, the achieved EOZ for an intended 6.0 mm OZ on an older generation VISX S2 for a − 12 D treatment, was similarly reported to be smaller at 14.5 mm^2^ [42].

Studies show that myopic excimer corneal ablations cause postoperative HOAs [26, 43–45], and these increase with greater myopic correction [43, 44, 46]. Postoperative total mean RMS ocular HOA was 1.07 ± 0.32 μm at 6.0 mm. This value is similar to that reported with the Alcon EX-500 with a lower myopia of SEQ of − 8.65 D (total RMS postoperative HOA: 1.24 ± 0.85 μm) [26]. Other studies with lesser myopia report a range from 0.37 μm to 1.24 μm, measured with various aberrometer technologies [47–50]. Similarly, ocular spherical aberrations (0.67 ± 0.25 μm) and ocular coma (0.70 ± 0.40 μm) were within the range of previously reported values for lesser myopia [47–50]. Of interest is that there were no increased visual phenomena, nor a perceived deterioration in subjective quality of vision at these levels of HOAs. As well, postoperative flat keratometry (mean Kmin: 33.0 D; range: 31.4 to 39.6 D) was not correlated with poorer subjective QoV (*P = 0.32*) nor patient satisfaction. Flat keratometry may not be a factor that contributes to safety or outcomes, in keeping with a recent publication [51]. Further studies on the effect of flat keratometry on QoV are needed.

A recent literature review on the satisfaction of modern LASIK outcome by Sandoval et al. [52] showed that the industry satisfaction rate is at 99%, and this included 97 studies that reported outcomes for patients that had low to high myopia. Previous studies have shown excellent subjective QoV and patient satisfaction in VHM eyes postoperatively [16, 53]. The current study is the first comparison of preoperative to postoperative subjective quality of vision (QoV) and patient satisfaction in VHM eyes. VHM LASIK did not induce additional subjective night vision disturbances compared to preoperative spectacle-corrected levels (Table 2) and significantly reduced the frequency of perceived glare (*P = 0.016*), starbursts (*P < 0.001*), and other visual phenomena (Table 2). Patients improved their preoperative spectacle-corrected subjective QoV score (7.5 to an uncorrected QoV of 9.1 postoperatively). Highly myopic contact lens intolerant and spectacle-dependent patients may have had noticeable NVDs and visual phenomena related to aberrations induced by their glasses preoperatively [37, 38]. Their quality of life may have also been hampered by the significant dependence on spectacles. Their perception of the benefit of surgery and being glasses free might be greater than a lesser myope or a contact lens wearer, thereby making their personality profile appropriate for LASIK. They may also be less demanding than those patients with lower myopia, and not looking for perfection. These patients were also counselled preoperatively regarding the high risk of regression, as well as the inability to treat a second time, leaving them with reasonable expectations. Even those patients with mild residual myopia after surgery rated their QoV as better, although as expected there was an inverse correlation between QoV rating and residual postoperative refractive error (*P = 0.02*). The residual myopia may have improved presbyopic symptoms as over half the patients were over age 40, and the induced positive spherical aberration could have also improved their near depth of field.

Although VHM patients may have appropriate personalities for LASIK with good potential for neural adaptation, surgeons should recognize that they require more chair time with detailed explanations regarding striae, ectasia, cataract, retinal detachment risk, and predictability of refractive outcomes should a future intraocular lens (IOL) be needed.

### Study limitations

Only 2 years of follow-up is presented. Longer follow-up is needed to see the true incidence of post VHM LASIK ectasia, and longer -term regression rates. This study did not measure preoperative HOAs and is unable to determine the change in HOAs induced by surgery. Only a random sampling of a third of patients received the subjective questionnaire and although the results were highly favorable these should be interpreted in that context. SMILE [54] and novel Phakic Intraocular lenses techniques [1] are all promising approaches that can be used to treat patients with high myopia. Further research is required to determine which treatment is optimal for specific patient groups and can create the least myopic regression.

## Conclusions

In summary, the WaveLight® Allegretto Wave® Eye-Q 400 Hz excimer laser, for VHM between − 10.00 D and − 13.50 D, results in better accuracy, efficacy and safety than those reported with previous generation lasers. Safety, accuracy, and efficacy at 2 years is comparable to lower levels of high myopia outcomes, while subjective uncorrected QoV is similar or better. Regression at the 2-year follow-up is very small, with excellent patient satisfaction, even in eyes with small residual myopia. Although longer term studies in these patients are needed, the results suggest that with appropriate patient screening, surgeons can consider broadening their LASIK candidacy parameters to include patients within this VHM group.

## Data Availability

All data generated or analyzed during the current study are included in this published article.

## Abbreviations

CDVA: Corrected distance visual acuity
CI: Correction index
D: Diopters
EOZ: Effective optical Zone
HOA: Higher-order aberrations
IOL: Intraocular lens
LASIK: Laser-Assisted in Situ Keratomileusis
LVC: Laser vision correction
MR: Manifest refraction
NVD: Night vision disturbance
PRK: Photorefractive keratectomy
PTA: Percent tissue altered
QoV: Quality of vision
RMS: Root mean square
RSB: Residual stromal bed
SD: Standard Deviation
SEQ: Spherical equivalent
UDVA: Uncorrected distance visual acuity
VHM: Very high myopia

## References

[1] Barsam A, Allan BD (2012). Excimer laser refractive surgery versus phakic intraocular lenses for the correction of moderate to high myopia. Cochrane Database Syst Rev.

[2] Huang D, Schallhorn SC, Sugar A, Farjo AA, Majmudar PA, Trattler WB, Tanzer DJ (2009). Phakic intraocular lens implantation for the correction of myopia: a report by the American Academy of ophthalmology. Ophthalmology.

[3] Wallerstein A, Caron-Cantin M, Gauvin M, Adiguzel E, Cohen M (2019). Primary topography-guided LASIK: refractive, visual, and subjective quality of vision outcomes for astigmatism 2.00 diopters. J Refract Surg.

[4] Wallerstein A, Gauvin M, Cohen M (2019). Effect of anterior corneal higher-order aberration ablation depth on primary topography-guided LASIK outcomes. J Refract Surg.

[5] Wallerstein A, Gauvin M, Qi SR, Bashour M, Cohen M (2019). Primary topography-guided LASIK: treating manifest refractive astigmatism versus topography-measured anterior corneal astigmatism. J Refract Surg.

[6] Wallerstein A, Gauvin M, Adiguzel E, Singh H, Gupta V, Harissi-Dagher M, Cohen M (2017). Clinically significant laser in situ keratomileusis flap striae. J Cataract Refract Surg.

[7] McAlinden C, Pesudovs K, Moore JE (2010). The development of an instrument to measure quality of vision: the quality of vision (QoV) questionnaire. Invest Ophthalmol Vis Sci.

[8] Kung JS, Manche EE (2016). Quality of vision after Wavefront-guided or Wavefront-optimized LASIK: a prospective randomized contralateral eye study. J Refract Surg.

[9] Reinstein DZ, Threlfall WB, Cook R, Cremonesi E, Sutton HF, Archer TJ, Gobbe M (2012). Short term LASIK outcomes using the Technolas 217C excimer laser and Hansatome microkeratome in 46,708 eyes treated between 1998 and 2001. Br J Ophthalmol.

[10] Li SM, Zhan S, Li SY, Peng XX, Hu J, Law HA, Wang NL (2016). Laser-assisted subepithelial keratectomy (LASEK) versus photorefractive keratectomy (PRK) for correction of myopia. Cochrane Database Syst Rev.

[11] Lundstrom M, Manning S, Barry P, Stenevi U, Henry Y, Rosen P (2015). The European registry of quality outcomes for cataract and refractive surgery (EUREQUO): a database study of trends in volumes, surgical techniques and outcomes of refractive surgery. Eye Vis (Lond).

[12] Pesudovs K (2005). Wavefront aberration outcomes of LASIK for high myopia and high hyperopia. J Refract Surg.

[13] Hashemi H, Miraftab M, Asgari S (2014). Comparison of the visual outcomes between PRK-MMC and phakic IOL implantation in high myopic patients. Eye (Lond).

[14] Artini W, BR S, Hutauruk JA, DG T, Kekalih A (2018). Predictive factors for successful high myopia treatment using high-frequency laser-in-situ Keratomileusis. Open Ophthalmol J.

[15] Orucoglu F, Kingham JD, Kendusim M, Ayoglu B, Toksu B, Goker S (2012). Laser in situ keratomileusis application for myopia over minus 14 diopter with long-term follow-up. Int Ophthalmol.

[16] Lindbohm N, Tuisku IS, Tervo TM (2009). LASIK for myopia of −9.00 to −17.00 D with the VISX STAR S2: 2- to 5-year follow-up. J Refract Surg.

[17] Rosman M, Alio JL, Ortiz D, Perez-Santonja JJ (2010). Comparison of LASIK and photorefractive keratectomy for myopia from −10.00 to −18.00 diopters 10 years after surgery. J Refract Surg.

[18] Vega-Estrada A, Alio JL. Femtosecond-assisted laser in situ keratomileusis for high myopia correction: long-term follow-up outcomes. Eur J Ophthalmol. 2019;1120672119834478:1–9. 10.1177/1120672119834478 30845834

[19] Xia LK, Ma J, Liu HN, Shi C, Huang Q (2018). Three-year results of small incision lenticule extraction and wavefront-guided femtosecond laser-assisted laser in situ keratomileusis for correction of high myopia and myopic astigmatism. Int J Ophthalmol.

[20] Low JR, Lim L, Koh JCW, Chua DKP, Rosman M (2018). Simultaneous accelerated corneal crosslinking and laser in situ Keratomileusis for the treatment of high myopia in Asian eyes. Open Ophthalmol J.

[21] Niparugs M, Tananuvat N, Chaidaroon W, Tangmonkongvoragul C, Ausayakhun S. Outcomes of LASIK for Myopia or Myopic Astigmatism Correction with the FS200 Femtosecond Laser and EX500 Excimer Laser Platform. Open Ophthalmol J. 2018;12:63–71. 10.2174/1874364101812010063. 10.2174/1874364101812010063 PMC5960747 29872485

[22] Liu YL, Tseng CC, Lin CP (2017). Visual performance after excimer laser photorefractive keratectomy for high myopia. Taiwan J Ophthalmol.

[23] Ikeda T, Shimizu K, Igarashi A, Kasahara S, Kamiya K (2017). Twelve-year follow-up of laser in situ Keratomileusis for moderate to high myopia. Biomed Res Int.

[24] Reinstein DZ, Carp GI, Archer TJ, Lewis TA, Gobbe M, Moore J, Moore T (2016). Long-term visual and refractive outcomes after LASIK for high myopia and astigmatism from −8.00 to −14.25 D. J Refract Surg.

[25] Hashemi H, Ghaffari R, Miraftab M, Asgari S. Femtosecond laser-assisted LASIK versus PRK for high myopia: comparison of 18-month visual acuity and quality. Int Ophthalmol. 2017;37(4):995–1001. 10.1007/s10792-016-0364-7. 10.1007/s10792-016-0364-7 27699605

[26] Hashemi H, Miraftab M, Ghaffari R, Asgari S (2016). Femtosecond-assisted LASIK versus PRK: comparison of 6-month visual acuity and quality outcome for high myopia. Eye Contact Lens.

[27] Ide T, Toda I, Fukumoto T, Watanabe J, Tsubota K (2014). Outcome of a 10-year follow-up of laser in situ laser keratomileusis for myopia and myopic astigmatism. Taiwan J Ophthalmol.

[28] Kanellopoulos AJ, Asimellis G (2013). Refractive and keratometric stability in high myopic LASIK with high-frequency femtosecond and excimer lasers. J Refract Surg.

[29] Alio JL, Vega-Estrada A, Pinero DP (2011). Laser-assisted in situ keratomileusis in high levels of myopia with the amaris excimer laser using optimized aspherical profiles. Am J Ophthalmol.

[30] Stonecipher KG, Kezirian GM, Stonecipher M (2010). LASIK for −6.00 to −12.00 D of myopia with up to 3.00 D of cylinder using the ALLEGRETTO WAVE: 3- and 6-month results with the 200- and 400-Hz platforms. J Refract Surg.

[31] Liu Z, Li Y, Cheng Z, Zhou F, Jiang H, Li J (2008). Seven-year follow-up of LASIK for moderate to severe myopia. J Refract Surg.

[32] Schallhorn S, Tanzer D, Sanders DR, Sanders ML (2007). Randomized prospective comparison of visian toric implantable collamer lens and conventional photorefractive keratectomy for moderate to high myopic astigmatism. J Refract Surg.

[33] Alio JL, Soria F, Abbouda A, Pena-Garcia P (2015). Laser in situ keratomileusis for −6.00 to −18.00 diopters of myopia and up to −5.00 diopters of astigmatism: 15-year follow-up. J Cataract Refract Surg.

[34] Lim SA, Park Y, Cheong YJ, Na KS, Joo CK (2016). Factors affecting long-term myopic regression after laser in situ Keratomileusis and laser-assisted subepithelial keratectomy for moderate myopia. Korean J Ophthalmol.

[35] Ogasawara K, Onodera T (2016). Residual stromal bed thickness correlates with regression of myopia after LASIK. Clin Ophthalmol.

[36] Torky MA, Alzafiri YA (2017). Visual and refractive outcomes of small-incision lenticule extraction in mild, moderate, and high myopia: six-month results. J Cataract Refract Surg.

[37] Roberts B, Athappilly G, Tinio B, Naikoo H, Asbell P (2006). Higher order aberrations induced by soft contact lenses in normal eyes with myopia. Eye Contact Lens.

[38] Tang CY, Charman WN (1992). Effects of monochromatic and chromatic oblique aberrations on visual performance during spectacle lens wear. Ophthalmic Physiol Opt.

[39] Santhiago MR, Smadja D, Gomes BF, Mello GR, Monteiro ML, Wilson SE, Randleman JB (2014). Association between the percent tissue altered and post-laser in situ keratomileusis ectasia in eyes with normal preoperative topography. Am J Ophthalmol.

[40] Saad A, Binder PS, Gatinel D (2017). Evaluation of the percentage tissue altered as a risk factor for developing post-laser in situ keratomileusis ectasia. J Cataract Refract Surg.

[41] Wallerstein A, Adiguzel E, Gauvin M, Mohammad-Shahi N, Cohen M (2017). Under-flap stromal bed CXL for early post-LASIK ectasia: a novel treatment technique. Clin Ophthalmol.

[42] Holladay JT, Janes JA (2002). Topographic changes in corneal asphericity and effective optical zone after laser in situ keratomileusis. J Cataract Refract Surg.

[43] Smadja D, Santhiago MR, Mello GR, Touboul D, Mrochen M, Krueger RR (2013). Corneal higher order aberrations after myopic wavefront-optimized ablation. J Refract Surg.

[44] Khan MS, Humayun S, Fawad A, Ishaq M, Arzoo S, Mashhadi F (2015). Effect of wavefront optimized LASIK on higher order aberrations in myopic patients. Pak J Med Sci.

[45] Jahadi Hosseini SH, Abtahi SM, Khalili MR (2016). Comparison of higher order aberrations after Wavefront-guided LASIK and PRK: one year follow-up results. J Ophthalmic Vis Res.

[46] Hu JR, Yan ZH, Liu CF, Huang LN (2004). Higher-order aberrations in myopic and astigmatism eyes. [Zhonghua yan ke za zhi] Chinese J Ophthalmol.

[47] Goyal JL, Garg A, Arora R, Jain P, Goel Y (2014). Comparative evaluation of higher-order aberrations and corneal asphericity between wavefront-guided and aspheric LASIK for myopia. J Refract Surg.

[48] Zhang J, Zhou YH, Li R, Tian L (2013). Visual performance after conventional LASIK and wavefront-guided LASIK with iris-registration: results at 1 year. Int J Ophthalmol.

[49] Gertnere J, Solomatin I, Sekundo W (2013). Refractive lenticule extraction (ReLEx flex) and wavefront-optimized Femto-LASIK: comparison of contrast sensitivity and high-order aberrations at 1 year. Graefes Arch Clin Exp Ophthalmol.

[50] Hashemi H, Ghaffari R, Miraftab M, Asgari S (2017). Femtosecond laser-assisted LASIK versus PRK for high myopia: comparison of 18-month visual acuity and quality. Int Ophthalmol.

[51] Janbatian H, Drake R, Melki S, Brenner J (2019). The effect of low predicted/calculated postoperative keratometry on corrected distance visual acuity after LASIK. J Cataract Refract Surg.

[52] Sandoval HP, Donnenfeld ED, Kohnen T, Lindstrom RL, Potvin R, Tremblay DM, Solomon KD (2016). Modern laser in situ keratomileusis outcomes. J Cataract Refract Surg.

[53] Schallhorn SC, Venter JA, Hannan SJ, Hettinger KA (2015). Outcomes of wavefront-guided laser in situ keratomileusis using a new-generation Hartmann-shack aberrometer in patients with high myopia. J Cataract Refract Surg.

[54] Qian Y, Chen X, Naidu RK, Zhou X (2020). Comparison of efficacy and visual outcomes after SMILE and FS-LASIK for the correction of high myopia with the sum of myopia and astigmatism from −10.00 to −14.00 dioptres. Acta Ophthalmol.

